# The Phytosterol Peniocerol Inhibits Cell Proliferation and Tumor Growth in a Colon Cancer Xenograft Model

**DOI:** 10.3389/fonc.2019.01341

**Published:** 2019-12-03

**Authors:** Beatriz del Carmen Couder-García, Nadia J. Jacobo-Herrera, Alejandro Zentella-Dehesa, Leticia Rocha-Zavaleta, Zaira Tavarez-Santamaría, Mariano Martínez-Vázquez

**Affiliations:** ^1^Departamento de Productos Naturales, Instituto de Química, Universidad Nacional Autónoma de Mexico, Coyoacán, Mexico; ^2^Unidad de Bioquímica, Instituto Nacional de Ciencias Médicas y Nutrición Salvador Zubirán, Mexico City, Mexico; ^3^Departamento de Medicina Genómica y Toxicología Ambiental & Programa Institucional de Cáncer de Mama, Instituto de Investigaciones Biomédicas, Universidad Nacional Autónoma de México, Coyoacán, Mexico; ^4^Departamento de Biología Molecular y Biotecnología, Instituto de Investigaciones Biomédicas, Universidad Nacional Autónoma de Mexico, Coyoacán, Mexico

**Keywords:** phytosterol, cytotoxicity, antiproliferative, antitumor, xenograft, apoptosis, colon cancer

## Abstract

**Objective:** This study aimed to evaluate the cytotoxic activity of peniocerol against human colon cancer cell lines and its antitumor effect *in vivo* in a xenograft model using *nu/nu* mice.

**Materials and Methods:** SW-620, HCT-15, and HCT-116 colon cancer cell lines were treated with peniocerol for cytotoxicity by crystal violet technique. Cell apoptosis induction was detected by flow cytometry, and the antitumor activity of peniocerol was evaluated in a xenograft model of HCT-116 in *nu/nu* mice. After treatment, the effect of peniocerol was analyzed in histological sections of tumors by immunohistochemistry using DAPI, anti-PCNA, and PARP-1 antibodies.

**Results:** Peniocerol inhibited cell growth and induced apoptosis *in vitro* in a time and dose-dependent manner. Besides, peniocerol administration (30 or 15 mg/kg) inhibited tumor growth and induced apoptosis in the xenograft mice. The lack of peniocerol toxicity was proved by a biochemical blood analysis of healthy *nu/nu* mice administrated with this sterol.

**Conclusions:** Our results proved that peniocerol induces apoptosis *in vitro* and *in vivo* assays.

## Introduction

Colorectal cancer (CC) is second cancer with the highest mortality rate worldwide, responsible for more than 880 thousand deaths, and is the third most common cancer, with almost 2 million incidents in both sexes and all ages (1). Although several chemotherapeutic options are available, 5-fluorouracil (5-FU) is still the base drug for the treatment of colorectal cancer in combination with other anticancer agents. For instance, the combination of oxaliplatin, 5-FU, and leucovorin is used after tumor surgery for patients undergoing treatment with curative intent for stage III (2). However, the drug with the desirable activity and adequate toxicity has not been developed yet. Therefore, searching for better antitumor drugs is not concluded. In this sense, the so-called natural products are a rich source of bioactive compounds that could be considered as prototypes against this cancer.

Phytosterols are plant sterols, structural components of the cell membrane that participate in the regulation of fluidity and permeability associated with the membrane (3). They are byproducts of a complex isoprenoid biosynthesis pathway through the squalene (4). Phytosterols belong to a family of more than 200 different compounds. The most common are β-sitosterol, campesterol, and stigmasterol (3). They have demonstrated protection against various chronic diseases such as cardiovascular, liver, diabetes, and different types of cancer (5–9). Cancer prevention studies show that a diet rich in phytosterols can reduce the risk of different types of cancer (10). For example, the intake of β-sitosterol, an anti-inflammatory agent (11), can prevent colon cancer (12, 13). In this context, we have previously published the anti-inflammatory activity *in vivo* and the cytotoxic activity *in vitro* against human breast and colon cancer cells of the peniocerol, a sterol isolated from the cactus *Myrtillocactus geometrizans* (Mart Ex Pfeiff) Console (14, 15). In this article, we show the apoptotic activity of the peniocerol both *in vitro* against the colon cancer cell line HCT-116, as well as its antitumor effect *in vivo*.

## Materials and Methods

### Extraction and Isolation

Peniocerol (3β, 6α-diol-cholest-8-ene) was isolated from *Myrtillocactus geometrizans* and purified as previously described (15). Copies of the original spectra are obtainable from the author.

### Cell Lines and Cell Culture

Colon carcinoma cells SW-620, HCT-15, and HCT-116 were obtained from the American Type Culture Collection (ATCC). Cells were cultured in proper media (DMEM, DMPQ8-1L) as previously reported.

### Animals

Male *nu/nu* mice, 6–8 weeks old were used. The animals were housed, fed, and maintained following the recommendations of the ethics committee. The Animal Research Committee approved the experimental procedures and were carried out in accordance to the Guidelines for the Care and Use of Animals of the Bioterium Laboratory of the Instituto Nacional de Ciencias Médicas y Nutrición Salvador Zubirán (INCMNSZ), Mexico City, Mexico.

### Solutions

Working solutions of peniocerol (25 mg/mL) were prepared in dimethyl sulfoxide (DMSO) (Sigma-Aldrich, St. Louis, MO, USA. Cat. D4540–100 mL) and stored at −20°C. The peniocerol dilutions (80, 40, 20, and 10 μM) were prepared with DMEM and the DMSO concentration was <0.2%. Cisplatin (Sigma-Aldrich, St. Louis, MO, USA. Cat. 479306-1G), was included as a positive control (1 mg/kg), dissolved in DMEM. For *in vivo* experiments, 25 mg/mL concentrated peniocerol solutions were prepared, dissolved in sesame oil and 5% DMSO and cisplatin 10 mg/kg in saline solution. All solutions were prepared on the day of administration.

### Cytotoxicity

The cells HCT-15, HCT-116, and SW-620 were seeded in 48-well-plates at a density of 4 × 10^4^ cells per cm^2^ in DMEM plus 10% FBS. The cells were incubated for 24 h in an atmosphere of 5% CO_2_ and 95% humidity at 37°C. After 24 h the cells were treated with serial concentrations of peniocerol (80, 40, 20, and 10 μM). Cell viability was evaluated at 24, 48, and 72 h. Cisplatin was used as a positive control at concentrations <10 μM. Medium plus DMSO was included as a negative control. After incubation, the cells were fixed with DMEM, 2% FBS, and 1.1% glutaraldehyde for 15 min at room temperature. Subsequently, the fixation medium was removed from the cells, allowed to dry, stained with 200 μL of violet crystal for 15 min, the violet crystal was removed and finally, the stained protein was solubilized with 500 μL of 10% acetic acid. Optical density values were determined at a wavelength of 595 nm. A dose-response curve was plotted for each compound and the IC_50_ was estimated using the Excel statistical program using linear regression. The tests were carried out in quadruplicate in three independent experiments.

### Flow Cytometric Detection of Apoptotic Cells

Apoptotic cell death was determined using flow cytometry with the identification of Annexin V and propidium iodide markers (GTX85591, GeneTex). HCT-116 colon cancer cells were seeded in 6-well-plates with a density of 1.5 × 10^5^ cells per well. The cells were incubated for 24 h in an atmosphere of 5% CO_2_ and 95% humidity at 37°C. After 24 h the cells were treated with vehicle, peniocerol (20 μM) and camptothecin (2 μM) as a positive control (each treatment was done in triplicate), at different times (24, 48, and 72 h). After incubation times, the cells were harvested with trypsin, washed with PBS and centrifuged at 1,500 rpm for 5 min. The cell aggregate was resuspended in 500 μL of binding buffer, then 5 μL of Annexin V-FITC, 5 μL of propidium iodide were added and finally incubated for 5 min, according to the manufacturer's instructions. The cells were analyzed using a FACS Can flow cytometer from the National Flow Cytometry Laboratory, 10,000 cells were analyzed with the BD Cell Quest Pro Software program.

### Toxicity Assessment in *nu/nu* Mice

Lethal dose 50 (LD_50_) determination was performed using two groups of three female mice each. One group was treated with 125 mg/kg and the second with 62.5 mg/kg of peniocerol. Food and water were administered up to 4 h after treatment. Mortality was observed during the first 4 h. The LD_50_ was determined by the formula LD_50_ = (M0 + M1)/2 where M0 is the dose that does not cause the death of any mouse in the group and M1 is the dose that causes the death of at least one mouse in the group (16). The determination of the doses that did not induce toxic effects was carried out using female *nu/nu* mice distributed in groups of three mice. Peniocerol was administered intraperitoneally (i.p.) in two treatment schemes, once a week, and three times a week both for 21 days (Figure 3A). The weight and behavior of the mice were monitored every third day during treatment. Mice were sacrificed on day twenty-one. Blood tests were performed in the Departamento de Patología de la Facultad de Medicina Veterinaria y Zootecnia, UNAM, Mexico City, México.

### Antitumor Activity Evaluation in Xenografted Mice

Male *nu/nu* mice were distributed in seven groups of 6 mice each. The animals were xenografted with 1.5 × 10^6^ HCT-116 cells resuspended in 100 μL of PBS and inoculated via subcutaneous in the right flank from the back of the animal. The treatments were administered (i.p.) in two schemes, once a week or three times a week, both for 21 days. The treatments started when the tumors reached a volume of 50 mm^3^. The groups were organized as follows:

1. Negative control: 5% DMSO dissolved in sesame oil, once a week.
2. Negative control: 5% DMSO dissolved in sesame oil, three times a week.
3. Positive control: cisplatin 4 mg/kg once a week.
4. Positive control: cisplatin 2 mg/kg, three times a week.
5. Peniocerol: 30 mg/kg once a week.
6. Peniocerol: 15 mg/kg once a week.
7. Peniocerol: 15 mg/kg three times per week.

The weight of the mice and the tumor growth were measured three times a week. Tumor volume was calculated using the formula V = π/6 × (larger diameter × [smaller diameter]^2^) (17). The experiment was carried out for 21 days, at the end of the experiment, the animals were weighed, euthanized and the tumors were extracted, fixed in formalin, and embedded in paraffin.

### Histologic Evaluation of Tumors

Paraffin-embedded tumors were cut in histologic sections of 5 μm thick and used for subsequent analysis with hematoxylin-eosin, DAPI, and immunohistochemistry. The samples were analyzed in an Olympus IX71 microscope, with the QImaging program at the Microscopy Unit of the Biomedical Research Institute, UNAM, Mexico.

#### Hematoxylin-Eosin Staining (H-E)

Tissue slides were exposed at a temperature of 60°C for 15–30 min and rehydrated with the following solution for 5 min: Xylol (twice), ethanol/xylol 50:50, 100% ethanol, 96% ethanol, 80% ethanol, 70% ethanol, 50% ethanol, distilled water, and PBS. The cell membrane was subsequently permeabilized with a 0.5% Triton solution x-100 for 30 min, followed by two washes with PBS of 5 min each. One hundred fifty microliter of hematoxylin was added to the sample for 2 min, washed with distilled water, and covered with eosin for 20 s, washed with distilled water and assembled.

#### 4′,6-Diamidino-2-Phenylindole (DAPI) Immunofluorescence

The dewaxing and rehydrated of tissues were performed as previously described for H-E staining. Then, the membrane was permeabilized with a 0.5% Triton solution x-100 for 30 min, followed by two washes with PBS of 5 min each. The kit used was Vectashield mounting medium for fluorescence with DAPI from Vector Laboratories.

#### Nuclear Antigen of Proliferation Cells (PCNA) Determination

The dewaxing and rehydrated of tissues were performed as previously described for H-E staining. The membrane was permeabilized with a 0.5% Triton solution x-100 for 30 min, followed by two washes with PBS of 5 min each. Antigen exposure was carried out heating the samples in a 0.25 mM sodium citrate solution at pH 6.2 for 20 min in a microwave oven. Then, samples were left at room temperature for 20 min and slides were washed twice with PBS for 5 min. Endogenous peroxidase was inhibited by exposing the tissues to 3% H_2_O_2_ for 30 min, followed by 2 washes with PBS for 5 min each. The cell membrane permeabilization was performed with a 0.5% Triton solution x-100 for 30 min, followed by two washes with PBS of 5 min each. Subsequently, the non-specific signal was blocked by incubation with 1% H_2_O_2_ + 5% albumin, in PBS for 30 min, followed by one wash with PBS of 5 min. The slides were incubated with the primary anti-PCNA antibody (sc-25280, Santa Cruz Biotechnology, INC). Incubation was performed overnight at 4°C, followed by two washes with PBS for 5 min. The slides were incubated with the secondary antibody for 60 min at 37°C (anti-mouse IgG for PCNA, GTX77315, GeneTex). The detection of immunohistochemical signals was performed with diaminobenzidine (DAB) for 10 min, the excess was removed with distilled water. Couterstain: Harris hematoxylin staining was performed for 3–5 min on each tissue, then the excess was removed with distilled water. It was covered with Li_2_CO_3_ for 15 s; then the excess was removed with distilled water. Once the samples dried completely, the assembly was carried out with EcoMount from Biocare Medical and covered with a coverslip. Qualitative analysis of PCNA positive cells was carried out using the ImageJ-win64 image processing package.

#### Poly(ADP-Ribose) Polymerase (PARP-1) Determination

The dewaxing and rehydrated of tissues were performed as previously described for H-E staining. The membrane was permeabilized with a 0.5% Triton solution x-100 for 30 min, followed by two washes with PBS of 5 min each. Antigen exposure was carried out, heating the samples in a 0.25 mM sodium citrate solution at pH 6.2 for 20 min in a microwave oven. Then, the samples were left at room temperature for 20 min, and slides were washed twice with PBS for 5 min. Endogenous peroxidase was inhibited by exposing the tissues to 3% H_2_O_2_ for 30 min, followed by two washes with PBS for 5 min each.

The cell membrane permeabilization was performed with a 0.5% Triton solution x-100 for 30 min, followed by two washes with PBS of 5 min each. Subsequently, the non-specific signal was blocked by incubation with 1% H_2_O_2_ + 5% albumin in PBS for 30 min, followed by one wash with PBS of 5 min. The slides were incubated with the primary anti-PARP-1 antibody (sc-8007, Santa Cruz Biotechnology, INC). Incubation was performed overnight at 4°C, followed by two washes with PBS for 5 min. The slides were incubated with the secondary antibody for 60 min at 37°C (anti-mouse IgG for PARP-1, GTX77315, GeneTex). The detection of immunohistochemical signals was performed with diaminobenzidine (DAB) for 10 min. The excess was removed with distilled water. Couterstain: Harris hematoxylin staining was performed for 3–5 min on each tissue, then the excess was removed with distilled water. It was covered with Li_2_CO_3_ for 15 s; then, the excess was removed with distilled water. Once the samples dried completely, the assembly was carried out with EcoMount from Biocare Medical and covered with a coverslip. Qualitative analysis of PARP-1 positive and claved-PARP-1 cells was carried out using the ImageJ-win64 image processing package.

### Data Analysis

Each experiment was performed in triplicate. Data are presented as mean ± standard deviation (SD) of three independent experiments. Statistical differences were determined using the GraphPad Prism 6.0 software program (GraphPad Software Inc., La Jolla, CA). Comparisons between the treated and control groups were made in the *t*-test of unpaired data and the two-way ANOVA. All comparisons are made concerning untreated controls. A statistical difference in ^*^P < 0.05 was considered.

## Results

### Cytotoxicity of Peniocerol

**Figure 1:**
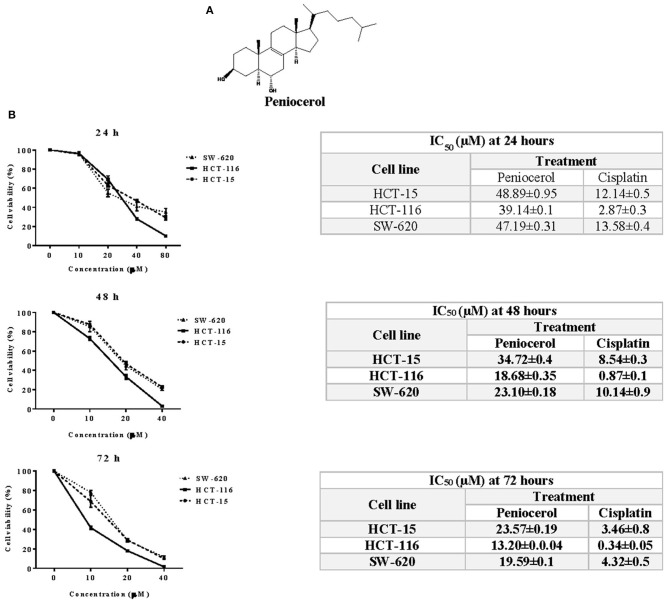
Cytotoxic activity of peniocerol in the colon cancer cell lines SW-620, HCT-116, and HCT-15. **(A)** Chemical structure of peniocerol. **(B)** Cell viability graphs represented in percentage with respect to the ascending results of peniocerol at different times, with their respective tables showing the average IC_50_ of each cell line, peniocerol and cisplatin as a positive control. The bars in the graphs represent the standard deviation of three independent experiments per quadruplicate and in the tables the averages ± SD of three independent experiments per quadruplicate are represented.

The chemical structure of peniocerol is shown in Figure 1A. The cytotoxic activity of peniocerol was assessed using the crystal violet cell staining technique. The HCT-116 cells were the most susceptible to peniocerol (Figure 1B), in comparison to the other two cell lines. Therefore, the HCT-116 line was used for subsequent experiments.

### Detection of Apoptosis

**Figure 2:**
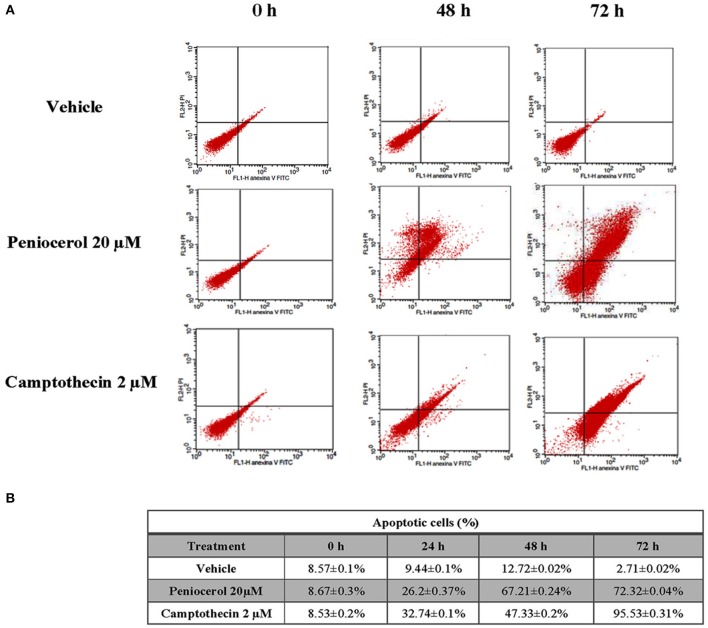
*In vitro* evaluation of apoptosis induction by peniocerol in the HCT-116 cell line. **(A)** Representative dot plots of HCT-116 cells treated with vehicle, peniocerol 20 μM and camptothecin 2 μM. **(B)** Table of percentages of early and late apoptosis induced by peniocerol. The numbers represent the average of three independent experiments ± SD.

HCT116 cells treated with peniocerol experimented apoptosis in a time-dependent manner. As shown in Figure 2A, cell death was observed after 48 h of treatment and increased after 72 h, 67.21 ± 0.24, and 72.32 ± 0.04%, respectively. The level of apoptosis induced by peniocerol was similar to that produced by camptothecin (Figure 2B). These results are totally in agreement with those previously obtained where it was proved, through cleavage of PARP-1 and flow cytometry, that this sterol induced apoptosis in several human cancer lines (14, 18).

### Toxicity of Peniocerol of Peniocerol Was Evaluated in Female *nu/nu* Mice, Using Two Different Schemes of Treatment

**Figure 3:**
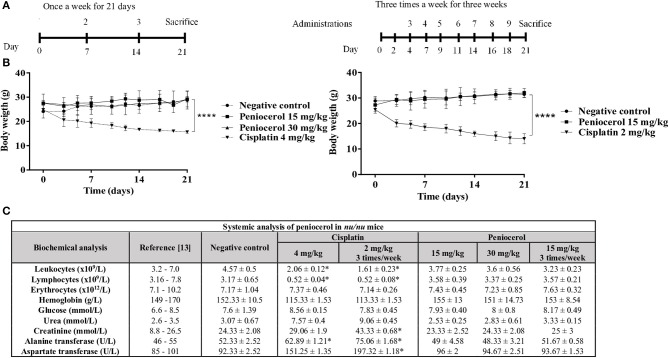
Toxicity evaluation of peniocerol in *nu/nu* mice. **(A)** Treatment scheme: Once a week for 3 weeks, the administrations were performed on days 0, 7, and 14; Three times a week for 3 weeks, administrations were performed, on days 0, 2, 4, 7, 9, 11, 14, 16, and 18. In both schemes, the animals were slaughtered on day 21. **(B)** Toxicity graphs: Once a week for 3 weeks, doses 30 mg/kg and 15 mg/kg of peniocerol, 4 mg/kg of cisplatin and as a negative control (sesame oil + DMSO 5%); three times a week for 3 weeks, doses 30 mg/kg and 15 mg/kg of peniocerol, 2 mg/kg of cisplatin and as a negative control the vehicle (sesame oil + 5% DMSO). The weight of the mice was monitored three times a week for 21 days. The results shown are the mean ± standard deviation of the monitoring of three mice. The significant difference ****p < 0.0001 was compared to the negative control (*t*-test). **(C)** Table of the systemic analysis of peniocerol in mice administered once a week and three times a week for 21 days. The data shown are the mean values ± SD of three mice per group. Significant difference *p < 0.05 compared to the vehicle (*t*-test).

We first evaluated the effect of peniocerol administrated once a week during 21 days at 15 or 30 mg/kg doses. Our results showed that no significant modification in body weight were noticed compare with those of animal control. As shown in Figure 3B, when mice received peniocerol three times a week at different doses, also no significant differences in weight were detected. In contrast, the administration of cisplatin one time a week produced a significant decrease in body weight compared with the negative control (*p* < 0.0001). The weight of mice treated with cisplatin was reduced by 42%, which suggests a toxic effect of the positive control. Administration of 15 mg/kg of peniocerol three times a week for 21 days did not produce a change in the weight of the animals as compared with the negative control. On the contrary, cisplatin administered in the lower dose (2 mg/kg) three times a week induced a significant decrease in body weight compared with the negative control (*p* < 0.0001).

To further analyze the potential toxic effect of peniocerol in healthy mice, we performed a biochemical blood analysis including glucose, hemoglobin, urea, creatinine, alanine transferase, and aspartate transferase, and a cell count including leukocytes, lymphocytes and erythrocytes (Figure 3C). The analysis revealed that peniocerol did not produce any change in blood biochemical and cellular components compared with those observed in mice that received the vehicle. In sharp contrast, administration of cisplatin resulted in a significant decrease in leukocytes, lymphocytes, and hemoglobin (*p* < 0.05) and an increase in the concentrations of liver enzymes alanine transferase and aspartate transferase (*p* < 0.05). These results suggest that peniocerol (15 mg/kg and 30 mg/kg) is less toxic than cisplatin, and that healthy mice tolerate it well.

### Anti-tumor Activity of Peniocerol

**Figure 4:**
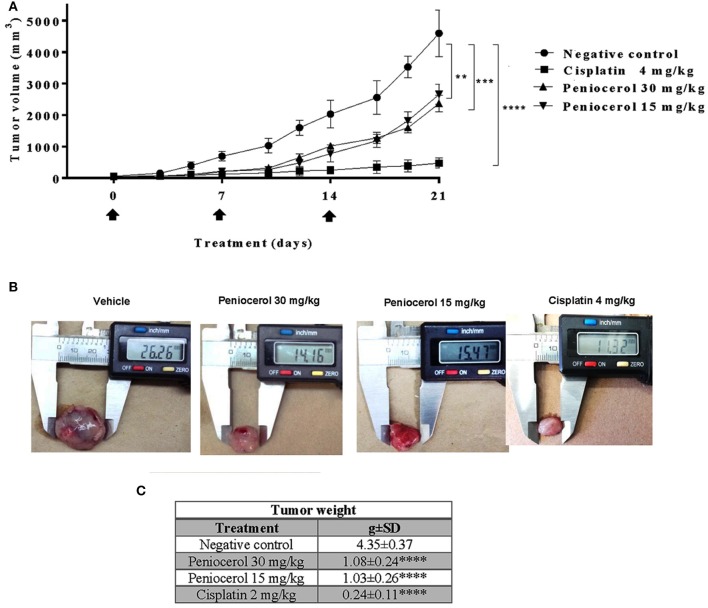
Antitumor activity evaluation of peniocerol once a week for 21 days administration in *nu/nu* mice. Treatment scheme: Once a week for 21 days, treatment started when the tumors reached a volume of approximate 50 mm^3^, on days 0, 7, and 14. The animals were sacrificed on day 21. **(A)** Antitumor activity graphs. Groups of six *nu/nu* mice inoculated with 1.5 × 10^6^ HCT-116 cells were treated once a week for 21 days with 30 mg/kg and 15 mg/kg of peniocerol, 4 mg/kg of cisplatin and the vehicle (sesame oil + 5% DMSO), on days 0, 7, and 14 (black arrows). The tumors were measured three times per week. The bars indicate the SD of the mean **p < 0.01, ***p < 0.001, and ****p < 0.0001 compared to the vehicle (ANOVA and *t*-test). **(B)** Photographs of the tumors at the end of the experiment. **(C)** Table of tumor weights ± SD at the end of the experiment.

To test the potential effect of peniocerol on tumor growth *in vivo*, we established a xenograft model in mice using the HCT-116 cell line. When the tumors reached a mean of 50 mm^3^ mice were treated with peniocerol (15 or 30 mg/kg doses) one time a week for 21 days, cisplatin (4 mg/kg) also one time a week for 21 days. As seen in Figure 4A, both concentrations of the peniocerol induced a significant reduction of tumor volume at the end of the experiment (*p* < 0.01). As expected, treatment with cisplatin-induced a highly significant reduction of tumor volume (*p* < 0.0001). At the finale of the experiment the remaining tumor masses were dissected and weighed, the results presented in Figures 4B,C demonstrated that peniocerol induced a reduction of tumor weight of 75.2 and 76.4% when administered at 30 or 15 mg/kg, respectively.

**Figure 5:**
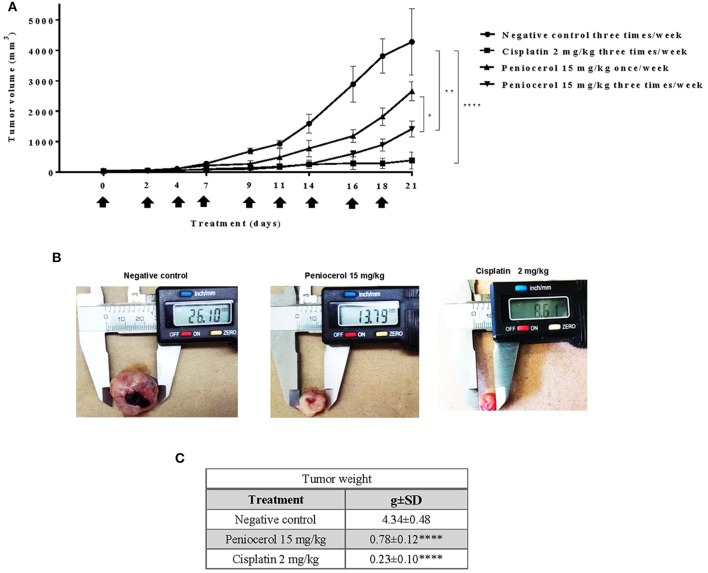
Antitumor activity evaluation of peniocerol three times a week for 21 day administration in *nu/nu* mice. Treatment scheme: Three times a week for 21 days, a total of nine administrations were performed, when the tumor reached an approximate volume of 50 mm^3^, on days 0, 2, 4, 7, 9, 11, 14, 16, and 18. The animals were sacrificed on day 21. **(A)** Antitumor activity graph. Groups of six *nu/nu* mice inoculated with 1.5 × 10^6^ HCT-116 cells were treated three times a week for 21 days, the doses tested were 30 mg/kg and 15 mg/kg of peniocerol, 2 mg/kg of cisplatin and as a negative control the vehicle was used (sesame oil + 5% DMSO). The size of the tumors was measured three times per week. The bars indicate the standard deviation of the mean *p < 0.01, **p < 0.001, and ****p < 0.0001 compared to the vehicle (ANOVA and *t*-test). **(B)** Photographs of the tumors at the end of the experiment. **(C)** Table of tumor weights ± SD at the end of the experiment.

Our results suggest that peniocerol has a significant anti-tumor effect on mice, so we decided to investigate whether an increase of frequency of administration of the lower doses evaluated of peniocerol would produce a major effect. Administration of peniocerol at 15 mg/kg doses three times a week for 21 days induced a significant decrement of tumor volume compared to that produced by the previously administration (*p* < 0.05; Figure 5A). However, it did not reach the level of inhibition produced by treatment with cisplatin (Figures 5B,C).

### Immunohistological Analysis of Tumors

**Figure 6:**
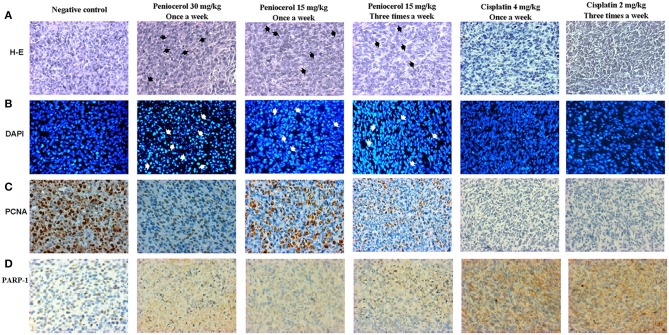
Tissues photomicrographs obtained from xenografted tumors with HCT-116 cells stained with; **(A)** hematoxylin-eosin (H-E) (Black arrows indicate amorphous and condensed shape). **(B)** 4′,6-diamidino-2-phenylindole (DAPI) (White arrows indicate condensation and fragmentation of the nuclei), **(C)** specific immunostaining for proliferating cell nuclear antigen (PCNA). The brown marks show the PCNA positive cells. **(D)** Specific immunostaining for poly(ADP-ribose) polymerase 1 (PARP-1) (brown nucleus) and Cleaved-PARP-1 (brown cytoplasm). The text at the top indicates the treatment for each experimental group. The acquisition of the images was done at 20x on the microscopic scale in the Q capture pro 5 QImaging capture software.

Tissue slides were obtained from tumors dissected after treatment. H-E showed a precise modification of cell morphology that can be observed by their amorphous and condensed shape in the tumor samples that were treated with peniocerol (30 and 15 mg/kg, once a week and 15 mg/kg three times a week), compared to the positive control (Figure 6A).

Furthermore, the nuclei tissues were stained with DAPI. As shown in Figure 6B, nuclei with condensation or fragmentation, indicative of apoptosis, can be seen in peniocerol treatments.

**Figure 7:**
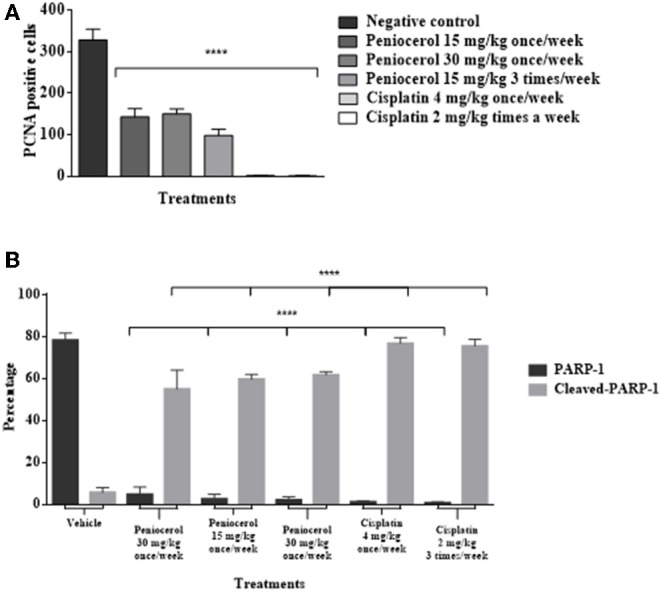
**(A)** Antiproliferative effect of peniocerol on the PCNA cell proliferation marker in the tissues of xenografted tumors of HCT-116 cells. **(B)** Apoptotic cell death effect of peniocerol on the PARP-1 and Claved-PARP-1 marker in the tissues of xenografted tumors of HCT-116 cells. The bars represent the average of positive cells ± standard deviation of three tissues analyzed by each experimental group. The data were analyzed and compared against the negative control ****p < 0.0001 (*t*-test).

To determine if peniocerol was able to inhibit cell proliferation, tumor slides were incubated with an anti-PCNA. Results of specific immunostaining indicate that both doses 30 and 15 mg/kg once a week and 15 mg/kg three times a week of peniocerol, decreased the number of positive cells compared to the negative control (Figure 6C). The results show that peniocerol significantly inhibited (*p* < 0.0001) the expression of PCNA at all three doses compared to the positive control. In the doses of 30 mg/kg and 15 mg/kg administered once a week, 150.07 ± 17.14 and 143.1 ± 34.7 labeled cells were detected respectively; while the dose of 15 mg/kg administered three times a week, 95.57 ± 18.85 cells were marked, compared with the negative control that was 328.3 ± 64.07 labeled cells (Figure 7A).

The induction of apoptosis in histological sections was determined with the specific immune staining of anti-PARP-1 (Figure 6D). The results show that independently of the peniocerol doses, there is a significant decrease (*p* < 0.0001) in the expression of PARP-1 in the nucleus, compared to the vehicle (78.34 ± 3.32%). On the contrary, in the expression of cleaved PARP-1, the doses of 30 mg/kg (54.96 ± 2.28%) and 15 mg/kg (59.96 ± 9.09%) once a week, and 15 mg/kg three times a week (61.7 ± 1.56%), significantly increase the percentage compared to the vehicle (5.64 ± 2.35%). Peniocerol treatments effected similarly to that of cisplatin (Figure 7B).

## Discussion

The National Cancer Institute (NCI, USA), has regulated the natural products antineoplastic activity to select potential compounds. Although it is not a formal rule, they are considered active when ED_50_ ≤ 4 μg/ml (19, 20). Under this premise, many compounds of natural origin, including phytosterols, would be considered inactive. However, there seems to be a probable relationship between the anti-inflammatory properties of some secondary metabolites such as the phytosterols, and their antitumor effects *in vivo* (21, 22). Our research group published the exceptional anti-inflammatory activity of peniocerol and its modest cytotoxic activity against the HCT-15 and the MCF-7 cancer cell lines *in vitro* (14). Consequently, we evaluated the antitumor activity of peniocerol in xenografted mice.

Although peniocerol showed cytotoxic activity on the colon cancer lines, HCT-15, HCT-116, and SW-620, the HCT-116 line was the most susceptible, so it was chosen for the next experiments. The level of apoptosis induced by peniocerol was similar to that produced by camptothecin *in vitro* (Figure 2B). In earlier studies, we proved that peniocerol might trigger both the caspase-dependent and caspase-independent apoptotic pathways (18).

To determine the toxicity of peniocerol female *nu/nu* mice were used. Our results showed that at 15 or 30 mg/kg doses of peniocerol under three or once a week administration, there was no significant modification in body weight compared with the untreated mice. It is worth to note that the cis-platin group showed until 42% of body weight loss (Figures 3A,B).

Biochemical blood analysis was performed to evaluate the potential effect of peniocerol in healthy animals. The analysis revealed that peniocerol did not produce any change in blood biochemical and cellular components compared with those observed in mice that received the vehicle. This analysis also reaffirms the toxicity so substantial that cisplatin induces in mice. For example, the administration of cisplatin resulted in a significant increase of alanine- and aspartate transferase as well as the decrease of hemoglobin, lymphocytes, and leukocytes. These results showed that in spite that both compounds showed similar antitumoral activity in the three times a week administration, their toxicity is quite different (Figure 3C).

The results registered that a higher frequency of administration of peniocerol improved its antitumor activity (Figure 5) and could suggest that the frequency of administration is more significant than the dose. Several studies have shown that dietary intake of phytosterols reduces the risk of suffering from diverse types of cancer. In experimental studies *in vivo* of ovarian, breast, colon and others neoplasia, was observed that the consumption of β-sitosterol or mixed phytosterols in diet, reduced the number of animals with tumors, or reduced the size of tumors (23–28). In epidemiological studies, the intake of β-sitosterol and stigmasterol was associated with lower risks of esophageal (29) and ovarian cancers (30), respectively. Moreover, it was reported that in female populations with minimal risk of breast cancer have a greater consume of phytosterols in the diet than those at high risk (31, 32).

Our findings show that the peniocerol tumor growth inhibition is related to an antiproliferative effect and induction of apoptosis. The PCNA is used in clinics as a classic marker of cell proliferation as a diagnostic and prognostic tool (33). PARP-1 is an abundant and ubiquitous nuclear enzyme related to DNA repair (34); its overexpression is linked to the development of some types of cancer. Therefore, PARP-1 inhibition selectively ends several types of tumorigenic cells (35). The significant decrease in the expression of PCNA in the tumor samples shows a condensation and fragmentation of the nuclei, thus an antiproliferative action (Figures 6C, 7A). Moreover, a significant decrease of PARP-1 in the nucleus and a significant increase of cleaved PARP-1 in the cytoplasm (Figures 6D, 7B) indicates apoptosis cell death.

In summary, the administration of peniocerol with a higher frequency, but with a lower dose provides a greater therapeutic effect, suggesting the possibility to develop an anticancer drug from this phytosterol.

## Data Availability Statement

Most of the data (Figures 1–7) used to support the findings of this study are included within the article. Copies of the peniocerol spectra are available upon request to the responsible author.

## Ethics Statement

The Animal Research Committee approved the experimental procedures and were carried out in accordance to the Guidelines for the Care and Use of Animals of the Bioterium Laboratory of the National Institute of Medical Sciences and Nutrition Salvador Zubirán, México.

## Author Contributions

MM-V contributed to the conception, writing, and discussion of the article. BC-G, contributed in all the experiments that were performed as well as in the discussion of the article. NJ-H contributed substantially in the conduct of animal experiments as well as in the writing and discussion of the manuscript. LR-Z contributed to the conduct of molecular biology experiments as well as the writing and discussion of the article. ZT-S contributed to the performance of some of the experiments. AZ-D contributed significantly to the discussion on molecular biology of the article. All authors discussed, reviewed, and approved the final version of the manuscript to be published.

### Conflict of Interest

The authors declare that the research was conducted in the absence of any commercial or financial relationships that could be construed as a potential conflict of interest.
